# Targeted Metabolomic Profiling of Total Fatty Acids in Human Plasma by Liquid Chromatography-Tandem Mass Spectrometry

**DOI:** 10.3390/metabo10100400

**Published:** 2020-10-09

**Authors:** Anas Al Aidaros, Charu Sharma, Claus-Dieter Langhans, Jürgen G. Okun, Georg F. Hoffmann, Majed Dasouki, Pranesh Chakraborty, Fatma Aljasmi, Osama Y. Al-Dirbashi

**Affiliations:** 1 Department of Genetics & Genomics, College of Medicine and Health Sciences, United Arab Emirates University, Al-Ain 17666, UAE; a.aidaros@uaeu.ac.ae (A.A.A.); aljasmif@uaeu.ac.ae (F.A.); 2 Department of Internal Medicine, College of Medicine and Health Sciences, United Arab Emirates University, Al-Ain 17666, UAE; charusharma@uaeu.ac.ae; 3 Department of General Pediatrics, Division of Neuropediatrics and Metabolic Medicine, Center for Pediatric and Adolescent Medicine, University Hospital Heidelberg, 69120 Heidelberg, Germany; claus-dieter.langhans@med.uni-heidelberg.de (C.-D.L.); JuergenGuenther.Okun@med.uni-heidelberg.de (J.G.O.); georg.hoffmann@med.uni-heidelberg.de (G.F.H.); 4 Department of Genetics, King Faisal Specialist Hospital and Research Center, Riyadh 3354, Saudi Arabia; madasouki@kfshrc.edu.sa; 5 Metabolics and Newborn Screening, Department of Pediatrics, Children’s Hospital of Eastern Ontario, Ottawa, ON K1H 8L1, Canada; pchakraborty@cheo.on.ca; 6 Department of Pediatrics, University of Ottawa, Ottawa, ON K1N 6N5, Canada; 7 Department of Pediatrics, College of Medicine and Health Sciences, United Arab Emirates University, Al-Ain 17666, UAE

**Keywords:** plasma fatty acids, targeted metabolomics, liquid chromatography-tandem mass spectrometry, derivatization, inborn errors of metabolism

## Abstract

This article reports a targeted metabolomic method for total plasma fatty acids (FAs) of clinical or nutritional relevance. Thirty-six saturated, unsaturated, or branched-chain FAs with a chain length of C8-C28 were quantified using reversed-phase liquid chromatography-tandem mass spectrometry. FAs in plasma (10 μL) were acid-hydrolyzed, extracted, and derivatized with DAABD-AE (4-[2-(*N*,*N*-Dimethylamino)ethylaminosulfonyl]-7-(2-aminoethylamino)-2,1,3-benzoxadiazole) at 60 °C for 1 h. Derivatization resulted in a staggering nine orders of magnitude higher sensitivity compared to underivatized analytes. FAs were measured by multiple-reaction monitoring using stable isotope internal standards. With physiological and pathological analyte levels in mind, linearity was established using spiked plasma. Intra-day (n = 15) and inter-day (n = 20) imprecisions expressed as variation coefficient were ≤10.2% with recovery ranging between 94.5–106.4%. Limits of detection and limit of quantitation ranged between 4.2–14.0 and 15.1–51.3 pmol per injection, respectively. Age-stratified reference intervals were established in four categories: <1 month, 1–12 month, 1–18 year, and >18 year. This method was assessed using samples from patients with disorders affecting FAs metabolism. For the first time, C28:0 and C28:0/C22:0 ratio were evaluated as novel disease biomarkers. This method can potentially be utilized in diagnosing patients with inborn errors of metabolism, chronic disease risk estimation, or nutritional applications.

## 1. Introduction

Fatty acids (FAs) are carboxyl group-containing compounds with a hydrocarbon chain of variable length and degree of unsaturation. Widely dispersed in nature, these organic compounds are often classified based on the number of carbon atoms as short (<6 carbons), medium (6–12 carbons), long (12–20 carbons), and very-long-chain (≥22 carbons) FAs. In addition to their remarkable role as fuel molecules, FAs are indispensable constituents of simple and complex lipids, such as triglycerides, phospholipids, and glycolipids, and their biological activities encompass signaling pathways, gene expression, and regulation of membrane structure and functions. These diverse functions substantiate the influence of proper FAs homeostasis on health, well-being, and risk of disease [1,2,3,4]. Disrupted FAs metabolism has been reported in association with several pathological conditions, including heart disease [5,6], cancer [7,8], insulin resistance, and non-insulin-dependent diabetes mellitus [9], Alzheimer neuropathology [10], and numerous inborn errors of metabolism [11,12].

The interest in FAs as biomarkers necessitates the availability of reliable analytical methods for quantitative and qualitative analysis in biological samples. Gas chromatography (GC) and gas chromatography-mass spectrometry (GC-MS) have been the primary analytical tools for FAs in all types of samples [13,14,15]. Analysis using these methods requires significant sample preparation that involves derivatization to enhance volatility, thermal stability, and chromatographic separation. Electrospray ionization tandem mass spectrometry (ESI-MS/MS) is a robust and versatile detection technique with established utilization in research and diagnostics [16]. With liquid chromatography (LC) as a front-end technology, LC-MS/MS methods represent powerful alternatives to GC and GC-MS due to the simpler workflow, better sensitivity, and faster analytical time [17]. Over the past years, applications of LC-MS/MS have expanded significantly in clinical laboratories in areas, such as therapeutic drug monitoring, drugs of abuse, clinical toxicology, and inborn errors of metabolism [18,19,20,21].

Although the analysis of native FAs by LC-MS/MS in the negative ion ESI mode is theoretically possible, in practice, this approach is often setback due to inefficient ionization and unpredictable fragmentation pattern [22,23]. Jemal et al., ascribed the suppression of FAs ionization to the inevitable use of acidic pH required for chromatographic resolution of these compounds by commonly used reversed-phase chromatographic systems [24]. To overcome this, we and others utilized carboxylic group derivatization to impart favorable chromatographic, ionization, and fragmentation properties of FAs [23,25,26,27,28,29,30,31,32,33]. Various derivatization reagents, including 2-nitrophenylhydrazine (2NPH) [32], dimethylaminoethanol, 3-picolylamine, or 3-pyridylcarbinol [26], N-(4-aminomethylphenyl) pyridinium [23,28], 2, 4-dimethoxy-6-piperazin-1-yl pyrimidine [27], 2,4-bis(diethylamino)-6-hydrazino-1,3,5-triazine [30], 4-[2-(N, N-dimethylamino) ethylaminosulfonyl]-7-(2-aminoethylamino)-2,1,3-benzoxadiazole (DAABD-AE) [25,31], and aminoxy TMT reagents [30] were utilized in these studies. Albeit chromatographic and mass spectrometric properties have been in general been improved, these methods were hampered by the long derivatization reaction time of 24 h [31], the additional demanding steps, such as liquid-liquid extraction to clean up the resultant derivatives [28,32], requirement of specific instrument configuration not commonly found in clinical laboratories [26,27], or the lack of diagnostic application and reference interval in human biological samples [23,28,29,30]. Further, methods which claimed clinical applicability did not address the analysis of diagnostically critical branched-chain FAs, such as phytanic acid (PHA; C20:0 branched), and pristanic acid (PRA; C19:0 branched), the primary pathognomonic markers of Refsum disease (RD) and α-methyl-CoA racemase deficiency [12,25].

Recently, Chen et al. described the analysis of FAs with broad chain length coverage of saturated C4:0-C26:0 as derivatives of 2-NPH by LC-MS/MS in the negative ion ESI mode [32]. While sensitive and reproducible, their method has major analytical flaws rendering it unsuitable for clinical applications. These include: (1) separation of linear and branched-chain isobaric C20:0 (i.e., arachidic acid and PHA) and isobaric C19:0 (i.e., nonadecanoic acid and PRA) has not been addressed, (2) the use of C19:0 as internal standard (IS) is inappropriate due to potential interference with PRA and invalidates the results of other FAs that utilize C19:0 as IS, (3) the reference interval of C26:0, the primary marker of Zellweger syndrome, reported by Chen et al. of 12.0 ± 5.7 µmol/L is concerning [32]. This value is significantly higher than the established reference interval in the literature and in clinical laboratories of ≤1.31 µmol/L and seems to be inaccurate, suggesting an unrecognized interference [14,25,34]. Further, reference intervals of other FAs, such as C8:0 and C10:0, reported by Chen et al., are orders of magnitude lower than known literature values [14] and should be reevaluated for potential analytical issues.

**Figure 1:**
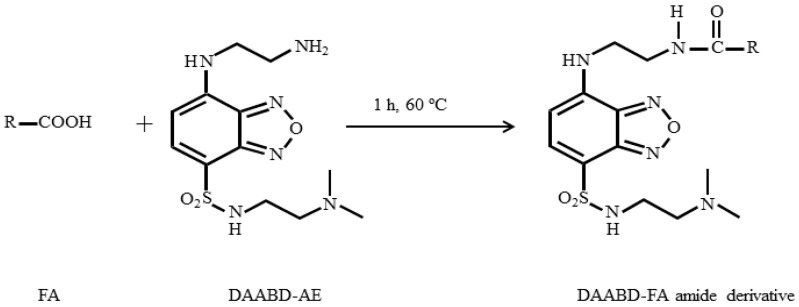
DAABD-fatty acids (FAs) amide derivative obtained by derivatizing FAs with DAABD-AE.

In the present study, we aimed at developing a high throughput quantitative method for FAs analysis for diagnostic and nutritional investigations using commonly available LC-MS/MS instrumentation. For this purpose, saturated, unsaturated, and branched-chain FAs with a chain length between C8 to C28 were analyzed after DAABD-AE derivatization (Figure 1). Where available, stable isotope-labeled analogs were used as IS. The method was optimized, validated, and applied to the analysis of total plasma FAs of healthy individuals and patients with established inborn errors of metabolisms.

## 2. Materials and Methods

### 2.1. Chemicals and Solvents

The following chemicals were purchased from Tokyo Chemical Industry (Tokyo, Japan): *n*-octanoic acid (C8:0), lauric acid (C12:0), myristic acid (C14:0), palmitic acid (C16:0), cis-9-hexadecenoic acid (C16:1), stearic acid (C18:0), γ-linolenic acid (C18:3), cis-5,8,11,14,17-eicosapentaenoic acid (EPA; C20:5) and arachidonic acid (C20:4). Decanoic acid (C10:0), 9-decenoic acid (C10:1), oleic acid (C18:1), arachidic acid (C20:0), all-cis-7,10,13,16,19-docosapentaenoic acid (DPA, C22:5), docosanoic acid (C22:0), tetracosanoic acid (C24:0), hexacosanoic acid (C26:0), DAABD-AE, N-(3-dimethylaminopropyl)-N′-ethylcarbodiimide hydrochloride (EDC), 4-(dimethylamino) pyridine (DMAP) and perfluorooctanoic acid (PFOA) were purchased from Sigma-Aldrich (Taufkirchen, Germany). The following deuterium or ^13^C labeled analogs used as IS were purchased from Cambridge Isotopes Laboratories (Tewksbury, MA, USA): ^13^C_4_-C8:0, d_3_-C10:0, d_3_-C12:0, d_3_-C14:0, d_4_-C16:0 and d_3_-C18:0. PRA, PHA, d_3_-PRA, d_3_-PHA, d_4_-C22:0, d_4_-C24:0, and d_4_-C26:0 were obtained from Dr. H. J. Ten Brink (Vrije Universiteit Medical Center, Amsterdam, The Netherlands). LC-MS/MS grade acetonitrile and water were purchased from Merck (Darmstadt, Germany). Merck also supplied us with HPLC grade hexane, toluene, and heptane.

### 2.2. Standard Solutions

Stock solutions of C16:0, C18:0, C18:1 and C20:4 at 30 mg/mL were prepared in toluene. Stock solutions of C12:0, C14:0, and C16:1 at 10 mg/mL were also prepared in toluene. Stock solutions (3 mg/mL) of C8:0, C10:0, C18:3 and C20:5 were prepared in toluene, whereas those of C22:0 and C24:0 were prepared in a mixture of toluene: heptane (1:1; *v/v*). Stock solutions (1 mg/mL) of C10:1, PRA, PHA, C20:0 and C22:5, ^13^C_4_-C8:0, d_3_-C10:0, d_3_-C12:0, d_3_-C14:0, d_4_-C16:0, d_3_-C18:0, d_3_-PRA, and d_3_-PHA were prepared in toluene, whereas those of C26:0, d_4_-C22:0, d_4_-C24:0 and d_4_-C26:0 were prepared in a mixture of toluene: heptane (1:1; *v/v*). These solutions were stored in tightly sealed amber glass screw-cap vials and were stable for at least six months at room temperature. Working solutions were prepared by diluting appropriate volumes in acetonitrile to produce the desired concentrations.

### 2.3. Study Samples

This study was approved by the Al Ain Medical District Human Research Ethics Committee (ERH-2017-555917-3). All experiments were carried out according to applicable local rules and regulations. Informed consent was obtained from participants or their parents and/or legal guardian for study participation.

The reference intervals of plasma total FAs were generated using samples collected from control subjects (n = 282). A commercially available software package (MedCalc version 19.4.1) was used to calculate double-sided 95 percentile reference intervals using the non-parametric percentile method. Plasma samples from patients with genetically confirmed inborn errors of metabolism were also analyzed (n = 18). Commercially available human plasma used for method development and optimization was purchased from BioIVT (Westbury, NY, USA). Except during use, samples were stored at −20 °C.

### 2.4. DAABD-AE Derivatization Reaction Optimization

We examined the concentration of DAABD-AE derivatization reagent and reaction time required to achieve optimal derivatization yield. Ten μL of standard FAs mixture containing C16:0 at 6000 μmol/L, C24:0 at 375 μmol/L, C8:0, C12:0, and C20:0 at 75 μmol/L each were placed in 100 × 13 mm screw-capped borosilicate tubes (Marienfeld, Germany) and evaporated to dryness under N_2_ gas. The residue was reconstituted in 200 μL of a mixture (1:1:2 *v/v/v*) of EDC (25 mmol/L in water), DMAP (25 mmol/L in acetonitrile), and DAABD-AE at different concentrations (2, 5, 7 or 9 mmol/L in acetonitrile). After incubation at 60 °C, the reaction was stopped with 2 mL of 10% acetonitrile in water containing 0.5 g/L PFOA (mobile phase A) at different time points (15, 30, 45, 60, 90, or 120 min). A portion of 1 μL of the resultant mixture was then subjected to LC-MS/MS analysis.

To assess sensitivity improvement obtained with DAABD-AE derivatization, we compared the signal to noise (S/N) ratio (n = 3) of C8:0, C12:0, C16:0, C20:0, and C24:0 with and without derivatization. These analytes were measured on the same LC-MS/MS system with optimized mass-to-charge (*m/z*) transitions, and identical mobile phase composition and injection volumes.

### 2.5. Sample Preparation

FAs were extracted from plasma as previously described [25] with slight modification. Briefly, 10 μL aliquots of plasma were transferred into 100 × 13 mm screw-capped borosilicate tubes and mixed with HCl (60 μL, 5.0 mol/L) and 400 μL of the working IS mixture (See Table 1 footnote for individual IS concentrations). The sealed tubes were then incubated at 100 °C for 1 h to release the bound FAs. After cooling to room temperature, the total FAs content was extracted by 1.0 mL of *n*-hexane through 3 min of vigorous shaking followed by centrifugation at 3800 rpm for 5 min at 4 °C. The hexane phase was transferred to a new borosilicate test tube and evaporated to dryness under a flow of N_2_ gas at room temperature.

DAABD-AE derivatization was achieved by reconstituting the extraction residue in 200 μL of a mixture (1:1:2 *v/v/v*) of EDC (25 mmol/L in water), DMAP (25 mmol/L in acetonitrile), and DAABD-AE (2 mmol/L in acetonitrile), followed by vortex mixing for 30 sec and incubation at 60 °C. After 60 min, 2.0 mL of 10% acetonitrile in water containing 0.5 g/L PFOA (mobile phase A) were added to stop the reaction. Aliquots of the resultant mixture (1 μL) were analyzed by LC-MS/MS.

### 2.6. LC-MS/MS System and Operating Conditions

Analyses were conducted on Shimadzu ultra-high-performance liquid chromatography (Nexera X2) consisting of two solvent delivery pumps, thermostated autosampler, column oven, degasser, and system controller (Shimadzu, Kyoto, Japan). An LC-MS 8060 triple quadrupole mass spectrometer equipped with ESI source operating in the positive mode was used for detection (Shimadzu). LabSolutions software (v 5.86; Shimadzu) running under Microsoft Windows 7 Professional environment was used to control the system and for data acquisition.

ESI-MS/MS analysis was achieved using N_2_ as nebulizing (3.0 L/min) and drying gas (10.0 L/min), whereas argon was used for collision-induced dissociation. Desolvation and ion source temperatures were set at 250 °C and 400 °C, respectively. The capillary voltage was +4.0 kV. Chromatographic separation was accomplished on a 2.1 × 50 mm, 1.7 μm C18 column maintained at 40 °C (Acquity UPLC BEH, Waters, Milford, CT, USA) using 10% acetonitrile in water containing 0.5 g/L PFOA (mobile phase A), and acetonitrile containing 0.5 g/L PFOA (mobile phase B). The gradient program involved varying the proportion of solvent B as follow: 0–1 min 40%, 1–3 min from 40% to 65%, 3–3.8 min 65%, 3.8–6 min from 65% to 88%, 6–8.5 min 88% and 8.5–11 min 95%. The column was re-equilibrated for 4 min using 40% mobile phase B. The flow rate was held at 0.35 mL/min. Table 1 specifies the analytical parameters employed in this work.

### 2.7. Method Validation

The linear relationship of analyte concentration *versus* detector response was assessed using plasma spiked with standard FAs to produce the concentration ranges shown in Table 1. Intra-day (n = 15) and inter-day (n = 20) imprecisions expressed as variation coefficient (CV%) were determined by repeated analysis of spiked plasma samples at two different levels. Analyte recovery was calculated using the following formula: Analyte recovery (%) = 100 × (measured concentration—endogenous concentration)/added concentration.

Limits of detection (LOD) were determined by recording the minimum concentrations that reliably produced S/N of 3. The limits of quantitation (LOQ) were calculated by establishing the analyte levels that produced S/N ratio of 10. Post-processing stability of DAABD-FA derivatives at 4°C was examined by repeatedly analyzing the reaction mixture of a plasma sample that was stored in the autosampler tray for 168 h (7 days) after sample preparation.

## 3. Results and Discussion

### 3.1. Derivatization of FAs with DAABD-AE

**Figure 2:**
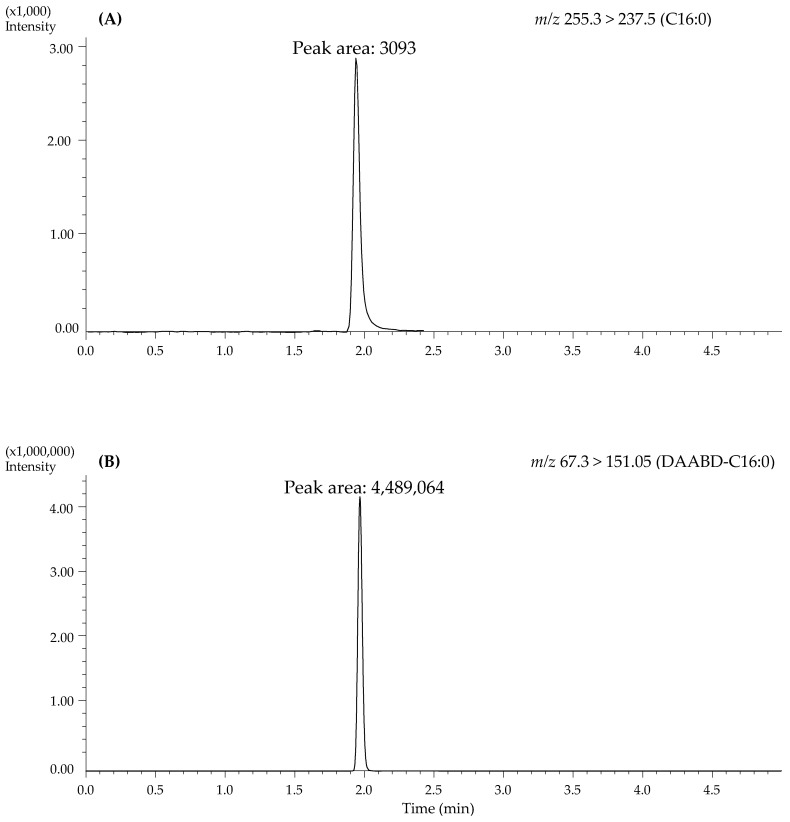
Extracted mass chromatograms for C16:0 free acid (2 µg on column) in negative ion mode at *m/z* 255.3 > 237.5 (**A**), and DAABD-C16:0 amide derivative (5 fg on column) in positive ion mode at *m/z* 567.3 > 151.1 (**B**).

In principle, analysis of unaltered FAs by LC-MS/MS can be achieved in the negative ESI mode using anion transitions generated from the elimination of water or carbon dioxide. In practice, neither of these transitions is adequately useful for reliable quantitation in complex matrices. This study aims to develop a simple, sensitive, and selective LC-MS/MS method to routinely quantify a broad range of FAs in small plasma volume for clinical evaluations. As shown in Figure 1, FAs were reacted with DAABD-AE to form stable amides with high proton affinity, ionization efficiency, and improved chromatographic properties. Collision-induced fragmentation produced a positively chargeable tertiary amine moiety with a mass-to-charge (*m/z*) ratio of 151originating from the derivatization reagent and was common to all studied analytes [25]. This *m/z* transition is detectable by positive ion ESI-MS/MS and was used conveniently to detect the studied FAs. In comparison with negative ion ESI-MS/MS detection of native FAs anions, the positive ion modification achieved through DAABD-AE derivatization resulted in significant improvement in detection sensitivity. To demonstrate the effect of derivatization on analytical sensitivity, we compared underivatized FAs with their DAABD-FA amides counterparts using the same LC-MS/MS system. Native FAs were analyzed under optimized conditions in the negative ESI mode, whereas DAABD-derivatives were analyzed by positive ESI. By comparing the S/N ratios normalized to the amount injected (µg), the sensitivity of DAABD-FA amides was a staggering nine orders of magnitude higher compared to native analytes irrespective of the FA chain length. This superior improvement of sensitivity determined a large number of FAs in a relatively small sample volume of 10 μL, an important consideration in the pediatric population. Figure 2 depicts representative chromatograms obtained with 2 μg of native C16:0 on column detected using *m/z* 255.3 > 237.5 (A) and 5 fg of DAABD-C16:0 on column detected at *m/z* 567.3 > 151.1 (B).

### 3.2. Method Development

#### 3.2.1. Derivatization of FAs with DAABD-AE

**Figure 3:**
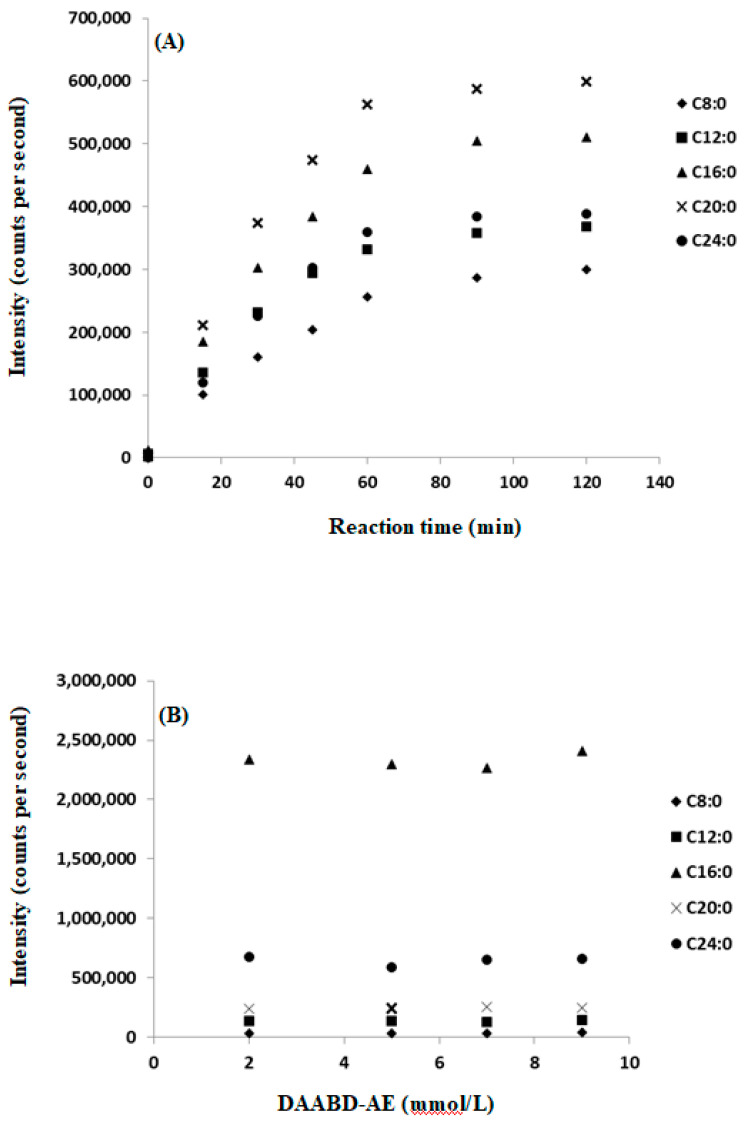
Derivatization yield of DAABD-FAs as a function of time (**A**), and the effect of DAABD-AE concentration on the derivatization yield (**B**).

The extraction of total FAs from a diminutive plasma volume (10 μL) was done as previously described [25]. Coupling of DAABD-AE with FAs was achieved using published conditions with minor modifications to accommodate the qualitative and quantitative diversity of analytes in this study [20,21,25,35,36]. This modification involved a facile single-step derivatization protocol that involves the use of premixed reagents added directly to the residual plasma extract followed by incubation at 60 °C for 1 h. When various DAABD-AE concentrations were tested, we confirmed that 2.0 mmol/L is adequate to achieve the desired derivatization yield. Figure 3A shows the derivatization yield of DAABD-FAs as a function of time, and Figure 3B illustrates the effect of DAABD-AE concentration on the derivatization yield. Predictably, derivatization with DAABD-AE imparted superb chromatographic, ionization, and fragmentation characteristic that allowed for multiplexed sensitive determination of a wide variety of clinically and nutritionally relevant FAs, including species at the extreme ends of the high and low abundance using 10 µL of plasma. In a recent work, Volpato et al. described that the derivatization of FAs with DAABD-AE can be achieved if the reaction mixture is incubated for 24 h at room temperature [31]. Our 1 h reaction conditions protocol is more practical than that of Volpato et al., as it allows for processing and reporting clinical samples without delay [31].

#### 3.2.2. Chromatographic Separation

**Figure 4:**
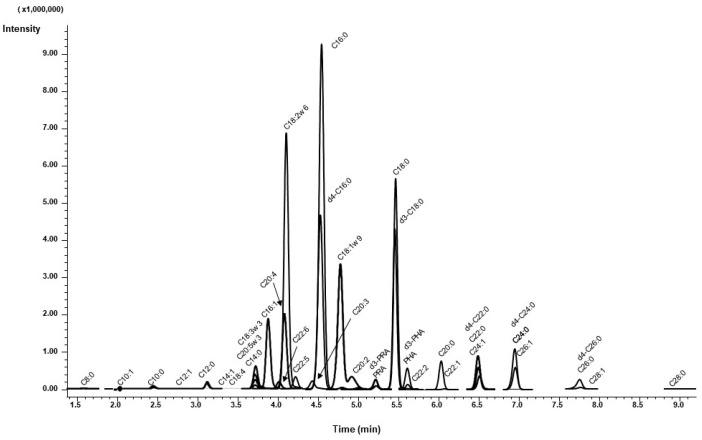
Representative multiple reaction monitoring LC-MS/MS, overlaid with chromatograms obtained by the current method.

Separation of DAABD-FAs by reversed-phase chromatography was achieved using a gradient program that increases the organic percentage of the mobile phase while maintaining constant ionic strength of the ion-pairing agent PFOA. Chemical standards and stable isotope IS were used for positive compound confirmation. FAs with shorter, branched, or unsaturated chains eluted faster than the longer, linear, or saturated FA compounds. Under the conditions used in this work, DAABD-C8:0 eluted first at 1.6 min, whereas that of DAABD-C28:0 eluted last at 9.1 min. Retention times for the studied FAs are shown in Table 1. With a column conditioning step, the injection-to-injection time was 15 min. This relatively short analysis time is an important consideration in high volume service labs, where competition on instrument time is high, and shorter analysis time is desirable. Figure 4 shows a representative multiple reaction monitoring LC-MS/MS, overlaid with chromatograms obtained by the current method.

#### 3.2.3. Linearity, LOD, and LOQ

Linearity was assessed using plasma samples spiked with commercially available standard FAs. The studied concentration ranges of FAs were selected to encompass physiological and pathological circumstances (Table 1). Compensation for potential analytical flaws was achieved by using appropriate stable isotope IS analogs. For compounds with no commercially available IS, the stable isotope analog with the nearest chain length was used (Table 1). Regression analysis by plotting the detector response of the analyte to IS ratio against the spiked concentration confirmed linear relationships (r ≥ 0.995) in the studied concentration ranges (Table 1). LOD and LOQ were established for analytes for which standard material is available commercially. As shown in Table 1, LOD (LOQ) expressed as pmol per injection ranged between 4.2 (14.0) for C16:0 and 15.1 (50.3) for C26:0. Despite that high sensitivity achieved in this work, the lower and higher limits of the dynamic range were selected to allow for reliable determination of normal and abnormal levels regardless of endogenous analyte abundance being at the high or the low end of the concentration spectrum.

#### 3.2.4. Imprecision and Recovery

Imprecision was evaluated by calculating the CV% of intra-day (n = 15), and inter-day (n = 20) studies using plasma spiked at two different FAs levels. With intra-day and inter-day CV% of less than 10.2% and 10.0%, respectively, the method described here is adequately reproducible (Table 2). The recovery of FAs calculated from spiked samples ranged from 94.5 to 106.4% (Table 2). DAABD-FA derivatives were stable for 72 h post-processing when kept in capped vials at 4 °C in the dark.

### 3.3. Determination of FAs Reference Intervals

In the present study, a total of 282 samples from control individuals were analyzed. Non-parametric double-sided 95 percentile reference intervals stratified according to age were established in four categories: Less than 1 month (n = 59), 1 to 12 months (n = 30), 1 to 18 years (n = 71), and more than 18 years (n = 122). Table 3 provides a summary of the reference intervals of total plasma FAs in μmol/L units obtained in this study.

Shown also are the reference intervals of the sum in mmol/L units of total FAs, saturated FAs, monounsaturated FAs (MUFA), and polyunsaturated FAs (PUFA). The reference intervals obtained in this study are comparable with those published in the literature [14].

### 3.4. Diagnostic Application on Samples from Patients with Inborn Errors of Metabolism

The diagnostic utility of the current method was evaluated using samples from patients (n = 18) with the following inborn errors of metabolism: Peroxisome biogenesis defect (PBD), X-linked adrenoleukodystrophy (X-ALD), adrenomyeloneuropathy (AMN), and RD. Results from five representative patients are shown in Table 4. In clinical laboratories, patients with PBD, X-ALD, and AMN are routinely diagnosed based on elevated plasma C26:0 and C26:0/C22:0 ratio. In this work, for the first time, we evaluated C28:0 and the C28:0/C22:0 ratio in these patients and observed significant elevations compared to controls (*p* < 0.0001). While C26:0 and its ratio to C22:0 are widely accepted as reliable diagnostic markers for peroxisomal disorders, C28:0 and its ratio to C22:0 described in this work are additional biomarkers with the potential to discriminate patients with PBDs from healthy individuals. This is of special importance in patients with subtle biochemical disruptions, such as patients 2, 4, and 5, shown in Table 4. Nonetheless, to establish C28:0 and its ratio to C22:0 as biomarkers of PBDs, additional studies are required to assess the diagnostic utility using a larger patients sample size that takes into account the clinical and genetic heterogeneity of PBDs. Interestingly, C28:0 and its C22:0 ratio was within the respective reference intervals in the patient with RD. This is not unexpected as this disorder is characterized by isolated PHA elevation due to deficiency of phytanoyl CoA hydroxylase, an enzyme not known to disrupt the peroxisomal β-oxidation pathway.

### 3.5. Method Comparison

**Figure 5:**
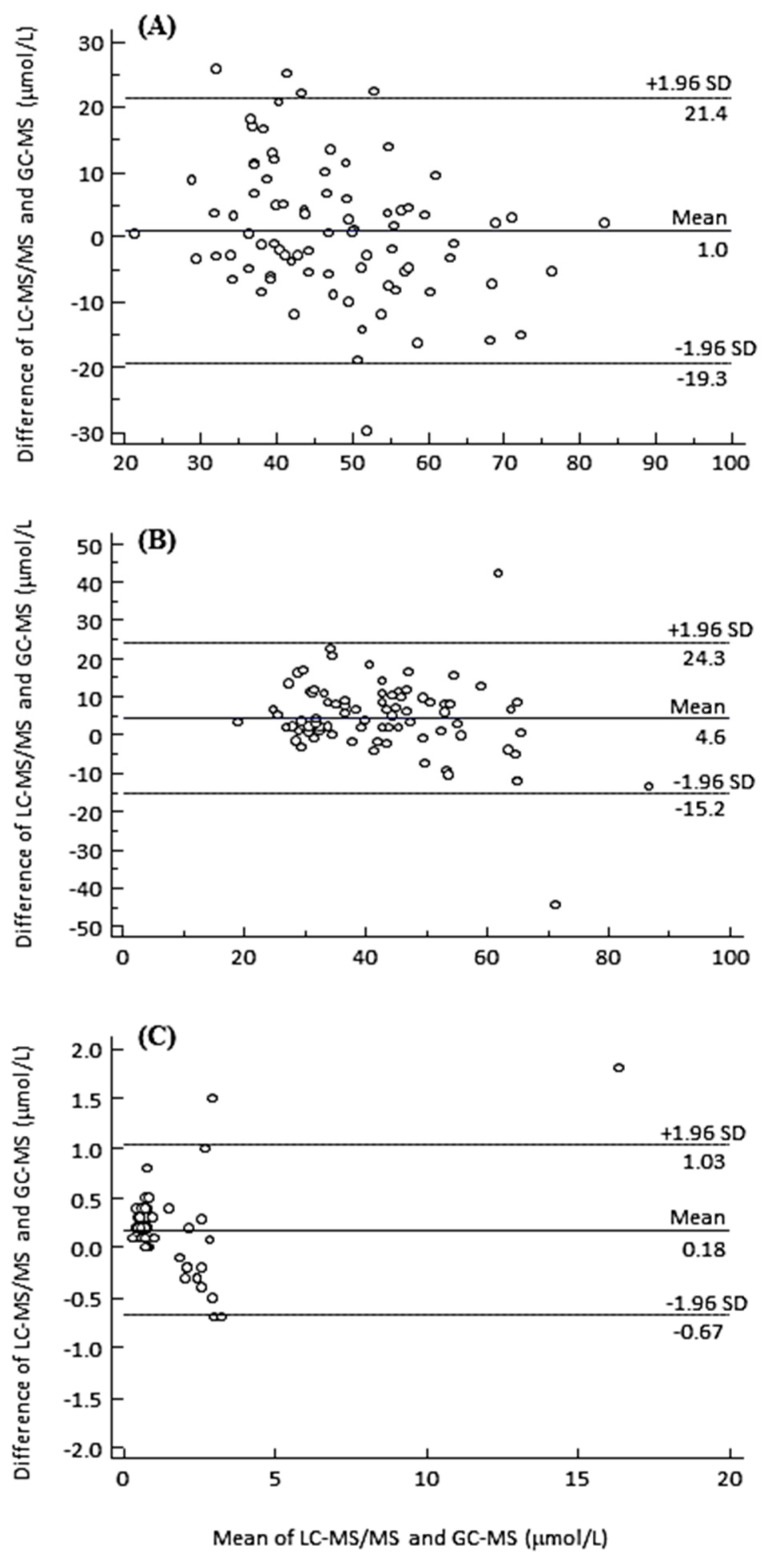
Bland-Altman plots for C22:0 (**A**), C24:0 (**B**), and C26:0 (**C**) obtained by the current LC-MS/MS method, and the routine GC-MS method. The *x*-axis shows the mean concentration, and the *y*-axis shows the difference in concentration of the two methods.

A group of FAs, namely, C22:0, C24:0, and C26:0 for which standard GC-MS methods are available, were used to demonstrate method comparison. These compounds are valued diagnostic markers for inborn errors of metabolism associated with peroxisomal dysfunctions. Plasma samples from patients with an established diagnosis of peroxisomal disease (n=18) and samples from unaffected individuals (n = 63) were used for comparison. Bland-Altman analysis suggests that the results obtained by the current method, which fall within the 95% confidence interval, are accurate and comparable to those obtained by gold-standard GC-MS (Figure 5).

Compared with other published LC-MS/MS methods for FAs [23,25,26,27,28,29,30,31,32,33], our method is superior because of the following: (1) Simultaneous analysis of 36 clinically relevant saturated, unsaturated, and branched-chain FAs species between C8-C28, (2) differentiation between diagnostically significant branched-chain FAs (i.e., PRA and PHA) and their linear-chain antipodes (C19:0 and C20:0), (3) establishment of age-specific reference intervals that are in agreement with the literature [14,34] technical simplicity (i.e., single-step derivatization with no need for derivatives clean up after reaction) that allows for high throughput routine analysis suitable for large volume service laboratories, and (4) utilization of standard LC-MS/MS instrumentation commonly found in clinical laboratories.

## 4. Conclusions

We have reported a new LC-MS/MS approach for the quantification of 36 FAs that range in chain length between C8 and C28. This approach utilizes the superior LC-MS/MS characteristics that DAABD-AE, as a derivatization reagent, imparts onto carboxylic acid compounds. Compared to native FAs analysis, DAABD-FA derivatization improved the detection sensitivity by nine orders of magnitude. This superb sensitivity allowed for carrying out this assay using as little as 10 μL of plasma with adequate precision and accuracy, as shown by method comparison with GC-MS. Our method offers equally high coverage for medium-, long-, and very-long-chain FAs that are clinically or nutritionally significant, including MUFA, PUFA, saturated, and branched-chain FAs. As such, it can potentially be utilized in the diagnosis and monitoring of patients with various inborn errors of metabolism, such as peroxisomal and mitochondrial FA oxidation, as well as defects involving arachidonic acid metabolism. In addition, circulatory FAs measured by our method may provide estimates of chronic disease risk (e.g., cardiovascular diseases and cancer), as well as providing guidance of appropriate dietary recommendations. Given the important clues on diagnostic hallmarks and dietary biomarkers it provides, we anticipate this method to find widespread utilization in clinical and nutritional applications.

## Figures and Tables

**Table 1:** Retention times, *m/z* of precursor ion, internal standard (IS), and linear range of studied FAs.

Compound	Retention Time (min)	IS *	Precursor Ion (m/z)	Linear Range (μmol/L)	LOD ^#^	LOQ ^§^
Octanoic acid, C8:0	1.6	(A)	455.3	0.75–75	9.9	33.0
Decenoic acid, C10:1	2.0	(B)	481.3	-		
Decanoic acid, C10:0	2.5	(B)	483.3	3.75–375	14.0	46.7
Lauroleic acid, C12:1	2.8	(C)	509.2	-		
Lauric acid, C12:0	3.2	(C)	511.2	7.5–750	12.7	42.3
Tetradecadienoic acid, C14:2	3.0	(D)	535.4	-		
Myristoleic acid, C14:1	3.4	(D)	537.4	-		
Myristic acid, C14:0	3.8	(D)	539.4	7.5–750	9.0	30.0
Hexadecenoic acid, C16:1w9	3.9	(E)	565.4	7.5–750	8.6	28.7
Palmitic acid, C16:0	4.7	(E)	567.3	60–6000	4.2	14.0
Stearidonic acid, C18:4	3.5	(F)	587.4	-		
α-Linolenic acid, C18:3w3	3.8	(F)	589.4	3.75–375	5.1	17.0
Linoleic acid, C18:2w6	4.2	(F)	591.4	-		
Oleic acid, C18:1w9	4.9	(F)	593.4	-	5.9	19.7
Stearic acid, C18:0	5.5	(F)	595.4	30–3000	6.8	22.7
EPA, C20:5w3	3.8	(H)	613.4	1.9–188	14.0	46.7
Arachidonic acid, C20:4w6	4.2	(H)	615.4	22.5–2250	8.6	28.7
h-γ-Linolenic acid, C20:3w6	4.5	(H)	617.4	-		
Eicosadienoic acid, C20:2	5.1	(H)	619.4	-		
Gondoic acid, C20:1	5.6	(H)	621.4	-		
Arachidic acid, C20:0	6.1	(H)	623.4	0.75–75	9.0	30.0
**Pristanic acid, C19:0 branched**	5.3	(G)	609.2	0.75–75	11.4	38.0
**Phytanic acid, C20:0 branched**	5.7	(H)	623.2	0.75–75	9.6	32.0
DHA, C22:6w3	4.1	(I)	639.2	-		
DPA, C22:5w3	4.6	(I)	641.2	0.75–75	14.3	47.7
DTA, C22:4w6	4.9	(I)	643.2	-		
Docosatrienoic acid, C22:3	5.5	(I)	645.2	-		
Docosadienoic acid, C22:2	5.8	(I)	647.2	-		
Docosenoic acid, C22:1	6.2	(I)	649.2	-		
Docosanoic acid, C22:0	6.6	(I)	651.2	3.75–375	10.3	34.3
Nervonic acid, C24:1	6.6	(J)	677.3	-		
Tetracosanoic acid, C24:0	7.1	(J)	679.3	3.75–375	9.8	32.7
Hexacosenoic acid, C26:1	7.1	(K)	705.3	-		
Hexacosanoic acid, C26:0	7.9	(K)	707.3	0.15–15	15.1	50.3
Octacosenoic acid, C28:1	7.9	(K)	733.3	-		
Montanic acid, C28:0	9.1	(K)	735.3	-		

* (A) ^13^C_4_ C8:0 at 7.5 μmol/L, (B) d_3_-C10 at 37.5 μmol/L, (C) d_3_-C12 at 75 μmol/L, (D) d_3_-C14 at 75 μmol/L, (E) d_4_-C16 at 600 μmol/L, (F) d_3_-C18 at 300 μmol/L, (G) d_3_-PRA at 7.5 μmol/L, (H) d_3_-PHA at 7.5 μmol/L, (I) d_4_-C22 at 37.5 μmol/L, (J) d_4_-C24 at 37.5 μmol/L, (K) d_4_-C26 at 1.5 μmol/L; ^#^ LOD, limit of detection (pmol/injection); ^§^ LOQ, limit of quantitation (pmol/injection).

**Table 2:** Recovery, intra-day, and inter-day precision of FAs were analyzed by the current method.

Compound	Sample	Concentration (μ mol/L)	Intra-Day (n = 15)			Inter-Day (n = 20)			Recovery (%)
			Mean	SD	CV (%)	Mean	SD	CV (%)	
C8:0	QC 1	38.0	36.6	2.4	6.6	36.3	0.6	1.8	98.7
	QC 2	72.4	73.4	2.5	3.4	73.4	2.5	3.4	
C10:0	QC 1	86.2	88.2	3.9	4.4	87.3	2.0	2.3	101.0
	QC 2	282.4	281.6	9.1	3.2	284.3	3.5	1.2	
C12:0	QC 1	209.7	201.7	7.9	3.9	200.9	3.7	1.8	98.5
	QC 2	580.5	588.7	11.7	2.0	584.9	5.3	0.9	
C14:0	QC 1	315.2	308.2	21.6	7.0	314.6	3.7	1.2	99.2
	QC 2	693.1	685.6	8.0	1.2	695.7	7.1	1.0	
C16:1	QC 1	406.6	397.8	25.0	6.3	398.5	3.9	1.0	98.8
	QC 2	675.7	671.9	28.8	4.3	675.7	8.8	1.3	
C16:0	QC 1	3663.1	3610.3	367.6	10.2	3697.7	46.0	1.2	99.5
	QC 2	5538.9	5620.3	213.7	3.8	5367.8	534.3	10.0	
C18:3w3	QC 1	124.8	129.9	7.5	5.8	127.6	8.5	6.7	100.8
	QC 2	330.8	325.6	21.9	6.7	324.8	3.8	1.2	
C18:0	QC 1	1655.4	1639.7	113.3	6.9	1640.6	35.1	2.1	99.4
	QC 2	2902.7	2889.7	39.8	1.4	2895.6	63.8	2.2	
C20:5w3	QC 1	94.9	94.6	2.5	2.6	95.8	2.5	2.6	101.1
	QC 2	177.2	181.7	6.5	3.6	179.7	2.4	1.3	
C20:4	QC 1	1340.2	1546.2	67.3	4.4	1621.3	145.4	9.0	106.4
	QC 2	2214.1	1965.4	41.8	2.1	2224.7	36.7	1.7	
C20:0	QC 1	32.5	33.5	0.4	1.3	31.2	1.3	4.3	94.5
	QC 2	75.4	67.8	2.0	2.9	67.0	2.5	3.7	
Pristanic	QC 1	16.6	16.9	0.6	3.4	16.4	1.0	6.2	98.5
	QC 2	55.9	55.1	2.3	4.2	53.4	2.0	3.8	
Phytanic	QC 1	16.8	17.0	1.0	5.7	16.3	0.4	2.3	98.0
	QC 2	56.2	55.0	2.0	3.6	54.1	1.5	2.8	
C22:5w6	QC 1	42.6	42.9	3.9	9.1	43.3	1.2	2.7	101.5
	QC 2	70.2	72.0	3.2	4.4	71.2	1.9	2.7	
C22:0	QC 1	124.9	124.0	2.0	1.6	123.3	1.4	1.2	99.2
	QC 2	317.7	317.5	12.6	4.0	314.4	5.1	1.6	
C24.0	QC 1	126.0	127.6	2.6	2.1	124.2	1.6	1.3	100.8
	QC 2	309.1	315.8	7.2	2.3	313.4	2.3	0.7	
C26.0	QC 1	3.8	3.7	0.1	3.0	3.7	0.1	1.5	97.8
	QC 2	11.8	11.4	0.2	1.9	11.7	0.2	1.5	

**Table 3:** Age-stratified reference intervals of total fatty acids in plasma (µmol/L).

Compound	<1 Month		1–12 Month		1–18 Year		>18 Year	
	Low	High	Low	High	Low	High	Low	High
Octanoic acid, C8:0	22	**53**	21	**60**	22	**62**	18	**41**
Decenoic acid, C10:1	0.2	**1.7**	0.2	**2.3**	0.1	**1.3**	0.1	**1.1**
Decanoic acid, C10:0	12	**46**	9	**60**	10	**57**	9	**41**
Lauroleic acid, C12:1	0.1	**4.0**	0.2	**2.8**	0.2	**1.6**	0.2	**2.4**
Lauric acid, C12:0	21	**165**	19	**211**	25	**202**	35	**152**
Tetradecadienoic acid, C14:2	0.1	**4.1**	0.2	**9.4**	0.2	**4.3**	0.1	**3.6**
Myristoleic acid, C14:1	0.7	**6.8**	0.8	**10.1**	1.0	**20.5**	1.2	**14.2**
Myristic acid, C14:0	35	**367**	43	**327**	37	**293**	40	**337**
Hexadecenoic acid, C16:1w9	163	**654**	74	**517**	49	**590**	72	**514**
Palmitic acid, C16:0	1304	**3654**	1289	**3595**	554	**3411**	1238	**3999**
Stearidonic acid, C18:4	0.0	**21.1**	0.1	**17.5**	0.2	**27.2**	0	**29**
α-Linolenic acid, C18:3w3	0.5	**46.9**	1.6	**66.8**	5.7	**62.7**	3	**44**
Linoleic acid, C18:2w6	216	**1750**	620	**2544**	655	**2193**	672	**2961**
Oleic acid, C18:1w9	925	**3250**	1237	**4943**	857	**4041**	816	**4433**
Stearic acid, C18:0	562	**1410**	580	**1553**	253	**1414**	511	**1507**
EPA, C20:5w3	10	**118**	7	**73**	9	**92**	6	**88**
Arachidonic acid, C20:4w6	622	**1652**	303	**1316**	122	**1155**	275	**1576**
h-γ-Linolenic acid, C20:3w6	30	**111**	17	**113**	29	**149**	23	**131**
Eicosadienoic acid, C20:2	4	**33**	5	**38**	5	**23**	5	**22**
Gondoic acid, C20:1	6	**36**	8	**49**	5	**38**	6	**35**
Arachidic acid, C20:0	11	**37**	5	**40**	5	**28**	5	**33**
Pristanic acid, C19:0 branched	1.1	**3.0**	1.1	**2.8**	1.2	**3.0**	1.3	**3.0**
Phytanic acid, C20:0 branched	1.6	**3.6**	1.8	**4.9**	1.7	**10.3**	1.8	**8.0**
DHA, C22:6w3	9	**60**	15	**64**	5	**45**	4	**39**
DPA, C22:5w3	13	**88**	7	**73**	8	**52**	4	**43**
DTA, C22:4w6	19	**68**	14	**64**	14	**53**	9	**61**
Docosatrienoic acid, C22:3	1	**7**	0	**5**	1	**6**	0	**4**
Docosadienoic acid, C22:2	3	**15**	3	**14**	2	**11**	2	**10**
Docosenoic acid, C22:1	15	**45**	14	**35**	12	**38**	11	**39**
Docosanoic acid, C22:0	27	**60**	21	**100**	28	**70**	28	**77**
Nervonic acid, C24:1	77	**257**	82	**220**	54	**210**	68	**267**
Tetracosanoic acid, C24:0	18	**59**	17	**70**	21	**65**	17	**76**
Hexacosenoic acid, C26:1	0.9	**3.2**	0.3	**2.0**	0.2	**1.9**	0.2	**1.9**
Hexacosanoic acid, C26:0	0.3	**1.0**	0.2	**1.0**	0.2	**1.2**	0.3	**1.1**
Octacosenoic acid, C28:1	0.01	**0.10**	0.00	**0.16**	0.00	**0.10**	0.01	**0.09**
Montanic acid, C28:0	0.07	**0.19**	0.04	**0.18**	0.03	**0.20**	0.03	**0.15**
Total fatty acids (mmol/L)	4.1	**14.1**	4.4	**16.2**	2.8	**14.4**	3.9	**16.6**
Total saturated fatty acids (mmol/L)	2.0	**5.9**	2.0	**6.0**	1.0	**5.6**	1.9	**6.3**
Total MUFA (mmol/L)	1.2	**4.3**	1.4	**5.8**	1.0	**4.9**	1.0	**5.3**
Total PUFA (mmol/L)	0.9	**4.0**	1.0	**4.4**	0.9	**3.9**	1.0	**5.0**

**Table 4:** Concentrations of relevant FAs in patients with peroxisomal disorders (μmol/L).

Patient	1	2	3	4	5	Reference Interval
Age	10 day	10.8 year	2.6 year	22.2 year	2 year	
Sex	F	M	F	M	F	
Diagnosis	Severe PBD	X-ALD	RD	AMN	Mild PBD	
**PRA**	1.6	1.9	3.7	1.7	4.2	1.2–3.0
**PHA**	4.3	4.3	23.2	4.7	15.2	1.7–10.3
**C22:0**	46.9	40.1	47.0	52.3	25.1	21–100
**C24:0**	63.8	57.0	28.0	86.7	24.5	17–76
**C26:0**	17.4	2.2	0.9	3.0	2.1	0.2–1.2
**C28:0**	1.82	0.38	0.19	0.38	0.20	0.03–0.2
**C24:0/C22:0**	1.36	1.42	0.76	1.66	0.98	≤1.20
**C26:0/C22:0**	0.372	0.054	0.019	0.057	0.082	≤0.022
**C28:0/C22:0**	0.0388	0.0094	0.0041	0.0073	0.0080	≤0.0045
